# *Himatanthus drasticus* Leaves: Chemical Characterization and Evaluation of Their Antimicrobial, Antibiofilm, Antiproliferative Activities

**DOI:** 10.3390/molecules22060910

**Published:** 2017-05-31

**Authors:** Cristiane Santos Silva e Silva Figueiredo, Joice Castelo Branco Santos, José Artur de Aguiar Castro Junior, Vinícius Galvão Wakui, João F. S. Rodrigues, Mariana Oliveira Arruda, Andrea de Souza Monteiro, Valério Monteiro-Neto, Maria Rosa Quaresma Bomfim, Lucília Kato, Luís Cláudio Nascimento da Silva, Marcos Augusto Grigolin Grisotto

**Affiliations:** 1 Programa de Pós-graduação em Biologia Parasitária, Universidade Ceuma, São Luís 65075120, Brazil; cristianeloud@gmail.com (C.S.S.S.F.); joic.cast@hotmail.com (J.C.B.S.); artur.aguiar.2008@hotmail.com (J.A.d.A.C.J.); joaofranciscosr@hotmail.com (J.F.S.R.); mariana_o.arruda@yahoo.com.br (M.O.A.); andreasmont@gmail.com (A.d.S.M.); valerio.monteiro@ceuma.br (V.M.-N.); mrqbomfim@gmail.com (M.R.Q.B.); 2 Laboratório de Produtos Naturais e Síntese, Instituto de Química, Universidade Federal de Goiás, Goiânia 74001-970, Brazil; vgwakui@gmail.com (V.G.W.); luciliakato@gmail.com (L.K.); 3 Centro de Ciências Biológicas e da Saúde, Universidade Federal do Maranhão, São Luís 65065545, Brazil; 4 Instituto Florence de Ensino Superior, São Luís 65020470, Brazil

**Keywords:** *Klebsiella pneumoniae*, inflammation, natural products, medicinal plants

## Abstract

Plant-derived products have played a fundamental role in the development of new therapeutic agents. This study aimed to analyze antimicrobial, antibiofilm, cytotoxicity and antiproliferative potentials of the extract and fractions from leaves of *Himatanthus* *drasticus*, a plant from the Apocynaceae family. After harvesting, *H. drasticus* leaves were macerated and a hydroalcoholic extract (HDHE) and fractions were prepared. Antimicrobial tests, such as agar-diffusion, Minimum Inhibitory Concentration (MIC) and Minimal Bactericidal Concentration (MBC) were carried out against several bacterial species. *Staphylococcus aureus, Pseudomonas aeruginosa*, *Listeria monocytogenes* and *Klebsiella pneumoniae* were inhibited by at least one extract or fraction in the agar-diffusion assay (inhibition halos from 12 mm to 30 mm). However, the lowest MIC value was found for HDHE against *K. pneumoniae*. In addition, HDHE and its fractions were able to inhibit biofilm formation at sub-inhibitory concentrations (780 µg/mL and 1.56 µg/mL). As the best activities were found for HDHE, we selected it for further assays. HDHE was able to increase ciprofloxacin (CIP) activity against *K. pneumoniae*, displaying synergistic (initial concentration CIP + HDHE: 2 µg/mL + 600 µg/mL and 2.5 µg/mL + 500 µg/mL) and additive effects (CIP + HDHE: 3 µg/mL + 400 µg/mL). This action seems to be associated with the alteration in bacterial membrane permeability induced by HDHE (as seen by propidium iodide labeling). This extract was non-toxic for red blood cell or human peripheral blood mononuclear cells (PBMCs). Additionally, it inhibited the lipopolysaccharide-induced proliferation of PBMCs. The following compounds were detected in HDHE using HPLC-ESI-MS analysis: plumieride, plumericin or isoplumericin, rutin, quercetin and derivatives, and chlorogenic acid. Based on these results we suggest that compounds from *H. drasticus* have antimicrobial and antibiofilm activities against *K. pneumoniae* and display low cytotoxicity and anti-proliferative action in PBMC stimulated with lipopolysaccharide.

## 1. Introduction

Infectious diseases are prevalent causes of morbidity and mortality in humans; particularly in tropical and developing countries like Brazil [1,2,3]. It is estimated that more than 2 million people are infected by bacterial pathogens each year, with hospitalized and/or immunodepressed individuals being the most affected. The bacteria frequently listed as etiological agent of nosocomial infections were gathered in a group acronymically named ESKAPE pathogens (*Enterococcus faecium*, *Staphylococcus aureus*, *Klebsiella pneumoniae*, *Acinetobacter baumannii*, *Pseudomonas aeruginosa* and *Enterobacter* spp.) [4]. Other bacteria species relevant for human infections are *Escherichia coli*, *Enterococcus faecalis* (commonly associated with urinary tract infections [5]), *Listeria monocytogenes* and *Salmonella enterica* (well-known foodborne pathogens [6]). These species are also highlighted due to their high ability to acquire antimicrobial resistance resulting in high levels of therapeutic failure [7].

Of special interest in this paper is the bacterium *K. pneumoniae*, which is a member of the ESKAPE group frequently reported as the etiologic agent of severe opportunistic infections and nosocomial outbreaks [1,2]. It is a Gram-negative bacillus, member of the Enterobacteriaceae family, that causes infections in the urinary tract, lower respiratory tract and surgical sites, septic arthritis, osteomyelitis and bacteremia [2,8,9,10,11,12,13,14]. *K. pneumoniae* usually forms mucoid colonies due to the production of a capsular polysaccharide (K antigen). This capsule is considered the most important virulence factor of *K. pneumoniae* that confers resistance to phagocytosis and prevents its death [8]. Biofilm formation is another known virulence property related to *K. pneumoniae* often associated with numerous cases of human severe infections [15]. Biofilms can be formed on the surfaces of the body, such as the teeth, skin, lung, bladder, catheters, endotracheal tubes, and joint implants [16]. *K. pneumoniae* biofilm is a particular problem in the hospital setting, where it can be found in endotracheal tubes used in newborns [13] and in urinary catheters [17]. In addition, several isolates of this species have the capacity to promote enzymatic inactivation of all β-lactams antibiotics, including carbapenems [10]. Carbapenemase-producing *K. pneumoniae* strains are important clinical challenge worldwide due to the limited antibiotic alternatives available to treat their infections [1,9]. Other molecular mechanisms are involved in the drug resistance of *K. pneumoniae*, for example resistance to ciprofloxacin have been attributed to overexpression of efflux pumps [18], mutations in DNA gyrase and topoisomerase IV [18,19], and production of siderophores [20]

We have reached a critical point in which new drugs are not being developed at the pace necessary to contain the natural ability of pathogens to acquire antibiotic resistance [7]. This scenario makes evident the need for constant search of new antimicrobial compounds and the development of treatment alternatives, such as, combinatorial therapy that uses one or more drugs that act on the same or different targets [21,22]. Therefore, the evaluation of the combined effect of drugs and phytochemicals has become common in prospecting programs for plant agents [23,24,25,26]. Another emerging approaches to fight microbial infections is the inhibition of the pathogen virulence factors [27] or the modulation of host’s immune system [28,29].

For a long time, popular medicine has represented an alternative to treat diseases in communities with limited access to modern medicines. In this context, plant-derived products play a fundamental role in the development of new therapeutic agents, due this empirical accumulation of ethnopharmacological knowledge over the years [30,31]. Another characteristic that favors the use of plant-derived products is their high chemical diversity, which could result in the inhibition/activation of molecular pathways different than those targeted by traditional drugs [32,33,34]. In this context, plants from the genus *Himatanthus* demonstrate pharmacologic potential [35]. For example, *Himatanthus drasticus* leaves (popularly known in Brazil as janaúba) are used by the population in Brazil to treat inflammation, gastritis, ulcers, and even cancer and infertility [36,37]. For this reason, this work aimed to evaluate the effectiveness of extracts and fractions of *Himatanthus drasticus* by analysis of their antimicrobial, antibiofilm and anti-proliferative potential. Furthermore, chemical characterization and cytotoxicity test were also performed. To the best of our knowledge, this is the first time that the effect of *Himatanthus drasticus* derived extracts on biofilm formation and on ciprofloxacin activity were evaluated.

## 2. Results

### 2.1. Antimicrobial Evaluation

Initially, a general evaluation of the antimicrobial activity of *H. drasticus* hydroalcoholic extract (HDHE) and its fractions was carried out against different species of bacteria using the agar diffusion assay (Table 1). In this assay, the HDHE was able to inhibit the growth of *L. monocytogenes* and *K. pneumoniae* with inhibition diameter zones (IDZs) of 12 ± 0.5 mm and 16 ± 0.5 mm, respectively. The hexane fraction (HDHF) inhibited the growth of *P. aeruginosa* (20 ± 0.5 mm), while the ethyl acetate fraction (HDEAF) inhibited *L. monocytogenes* (30 ± 0 mm). Finally, the butanolic fraction (HDBF) inhibited *S. aureus* (10 ± 0.5 mm) and *P. aeruginosa* (25 ± 0.5 mm).

In order to confirm the results obtained in the agar diffusion assay, we determined the MIC and MBC values for all extracts with positive results (Table 2). Interestingly, although HDEAF had the best result against *L. monocytogenes* in the diffusion assay, it was not possible to determine MIC for this pathogen (>50,000 µg/mL). In the other hand, the best antimicrobial potential was observed for HDHE with MIC and MBC values of 6250 µg/mL against *K. pneumoniae*. This same extract had both MIC and MBC of 25,000 µg/mL against *L. monocytogenes*. The MIC and MBC values for HDBF against *K. pneumoniae* were 12,500 µg/mL. For the other bacteria, MIC and MBC values were all higher than 50,000 µg/mL.

### 2.2. Antibiofilm Evaluation

**Figure 1:**
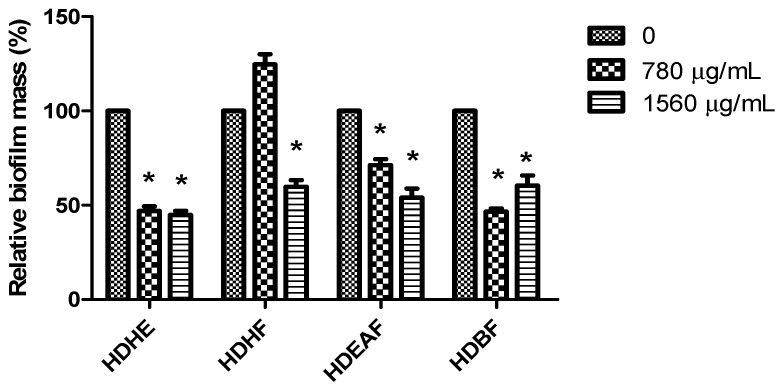
Inhibition of *Klebsiella. pneumoniae* biofilm by hydroalcoholic extract from leaves of *Himatanthus drasticus* and its fractions. HDHE: *H. drasticus* hydroalcoholic extract; HDHF: *H. drasticus* hexane fraction; HDEAF: *H. drasticus* ethyl acetate fraction; HDBF: *H. drasticus* butanolic fraction. * *p* < 0.05 in relation to untreated biofilm (control). The tests were performed in two independent assays in quadruplicate.

The action of HDHE and its fractions on the biofilm produced by *K. pneumoniae* was analyzed using sub-inhibitory concentrations (780 µg/mL and 1560 µg/mL). All samples were able to significantly inhibit the biofilm production after 24 h of treatment (*p* < 0.05), except for the hexane fraction at the concentration of 780 µg/mL (Figure 1). The best results were seen for treatment with 1560 µg/mL (*p* < 0.05), except for HDHE. Furthermore, the higher reduction in biofilm formation was achieved with HDHE fraction (approximately 50% for both 780 µg/mL and 1560 µg/mL). HDEAF and HDBF showed inhibitory values ranging from 28.73% to 53.4%. In this scenario, HDHE was chosen to be tested against *K. pneumoniae* for the remaining tests.

### 2.3. Combinatory Effects of Himatanthus drasticus Hydroalcoholic Extract and Ciprofloxacin against Klebsiella pneumoniae

**Figure 2:**
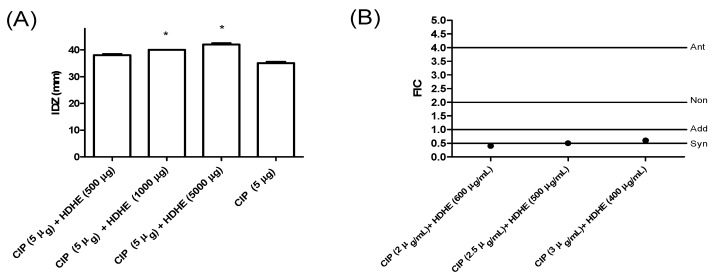
Combinatory effects of hydroalcoholic extract from leaves of *Himatanthus. drasticus* and ciprofloxacin against *Klebsiella. pneumoniae* evaluated by agar diffusion assay (**A**) and microdilution assay (**B**). HDHE: *H. drasticus* hydroalcoholic extract; CIP: ciprofloxacin; FIC: fractional inhibitory concentrations. * *p* < 0.05 in relation to ciprofloxacin disk. The tests were performed in two independent assay in quadruplicate.

To evaluate the effect of HDHE and its fractions on the action of ciprofloxacin, different amounts of each sample (500 µg, 1000 µg or 5000 µg) were added to disks containing 5 μg of the antibiotic. The amounts of extracts added in each disk were lower than those used in the initial diffusion test (10,000 µg). HDHE was able to significantly increase (*p* < 0.05) the inhibitory effect of ciprofloxacin when added at 1000 µg or 5000 µg (Figure 2A). The combined effect of the crude extract with ciprofloxacin against *K. pneumoniae* was confirmed by the determination of FIC. It was observed that two extract/drug combinations resulted in synergistic effects —initial concentrations (drug + extract) of 2.5 µg/mL + 500 µg/mL and 2 µg/mL + 600 µg/mL (FIC ≤ 0.5). The other combination resulted in additive effect (FIC = 0.6)—initial concentrations (drug + extract) of 3 µg/mL + 400 µg/mL—(Figure 2B).

### 2.4. Effect of Hydroalcoholic Extract of Himatanthus drasticus on the Integrity of the Bacterial Membrane

**Figure 3:**
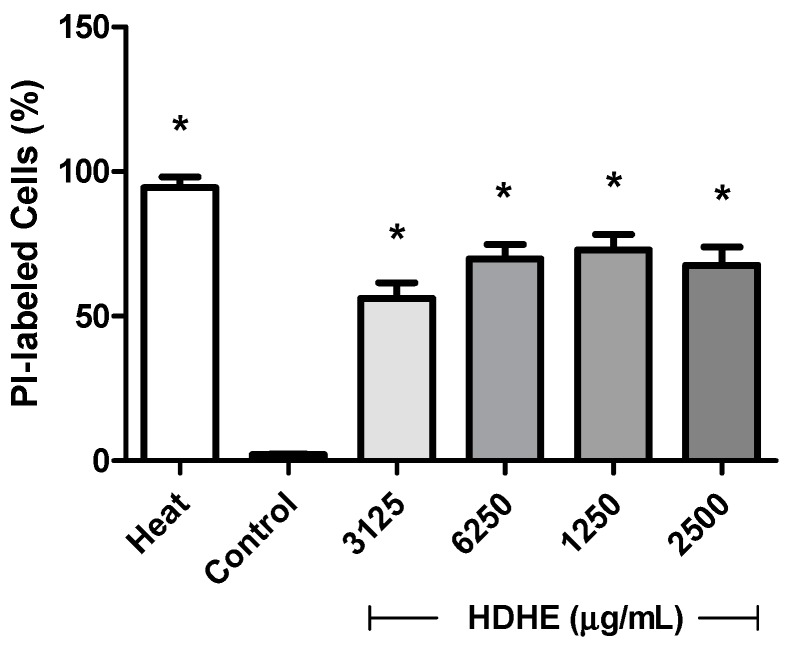
Effect of hydroalcoholic extract from leaves of *Himatanthus drasticus* on *Klebsiella. pneumoniae* membrane. Membrane disruption was assessed by Propidium Iodide (PI) incorporation indicating. HDHE: *H. drasticus* hydroalcoholic extract. * *p* < 0.05 in relation to untreated cells. The experiment we performed in two independent assays and at least 20,000 cells were analyzed.

To evaluate the action of HDHE on membrane integrity, the bacteria were treated with different concentrations of the extract (3125, 6250, 12,500 and 25,000 µg/mL) for 4 h and then stained with PI. Untreated cells (negative control) showed low fluorescence intensity indicating intact membrane (approximately 2.13 ± 0.18% of bacterial cells were labeled). On the other hand, the vast majority of bacteria submitted to thermal shock presented damage to the bacterial membrane, as evidenced by PI incorporation (94.4 ± 5.37% of the sample was labeled). After treatment with HDHE, the percentage of dead bacteria (PI+) increased in relation to untreated cells, (the percentage of PI+ cellsranged from 56.2 ± 7.50% to 72.9 ± 7.1%) (Figure 3). No statistically differences were found between the effects of tested HDHE concentrations.

### 2.5. Effect of Himatanthus drasticus Hydroalcoholic Extract on Cell Viability and LPS-Induced Proliferation of PBMCs

**Figure 4:**
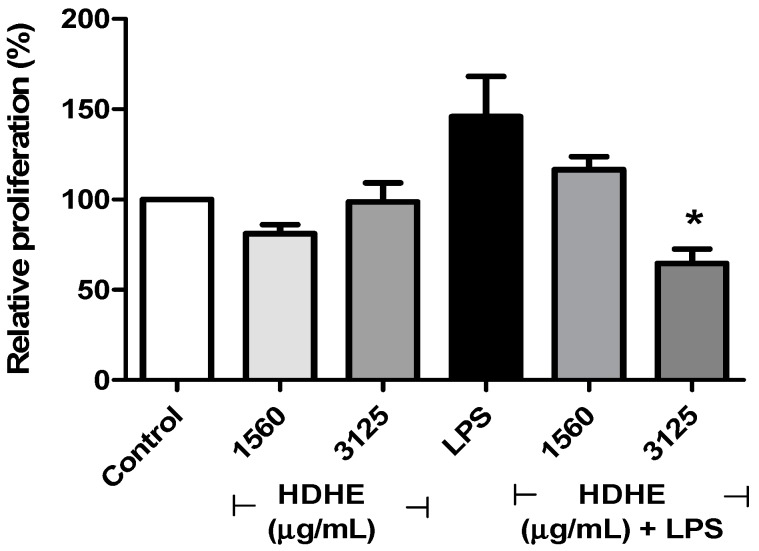
Effect of hydroalcoholic extract from leaves of *Himatanthus drasticus* on LPS-stimulated proliferation of human PBMC. HDHE: *H. drasticus* hydroalcoholic extract. * *p* < 0.05 in relation to LPS-treated PBMC (positive control). The experiment we performed in two independent assays and at least 20,000 cells were analyzed.

Cytotoxicity of HDHE was evaluated using human erythrocytes and PBMC. It was observed that the extract did not show significant hemolysis at the concentrations tested. Likewise, no significant changes were detected in viability or nitric oxide (NO) production by PBMC incubated with the extract for 24 h (data not shown). Since HDHE did not show significant toxic effects, we evaluated the proliferation of PBMC treated with HDHE in the presence or absence of LPS. After 72 h of cell culture, no significant changes were observed on the proliferation index in HDHE treated or control cells. On the other hand, LPS treated cells exhibited a higher proliferation rate, which was significantly inhibited by HDHE at the concentration of 3125 µg/mL (*p* < 0.05) (Figure 4).

### 2.6. Chemical Characterization of Himatanthus drasticus Hydroalcoholic Extract

**Figure 5:**
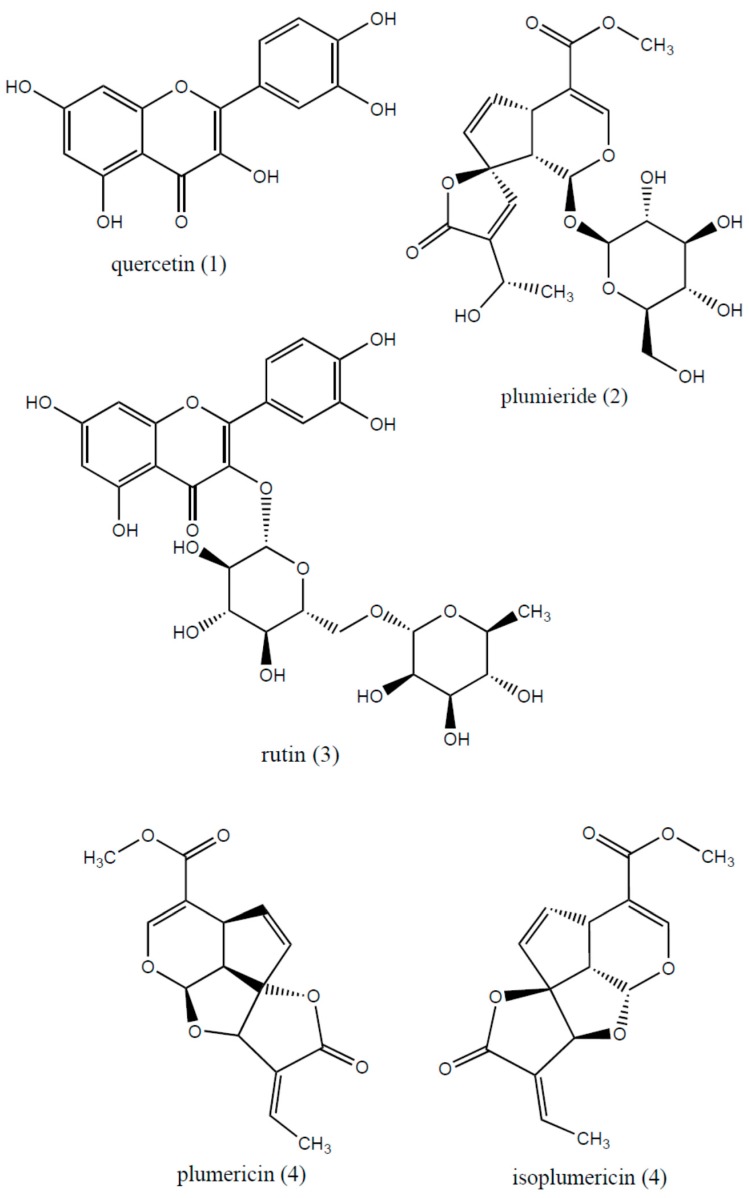
Chemical structure of compounds identified by HPLC-ESI-MS in hydroalcoholic extract from leaves of *Himatanthus drasticus*.

**Figure 6:**
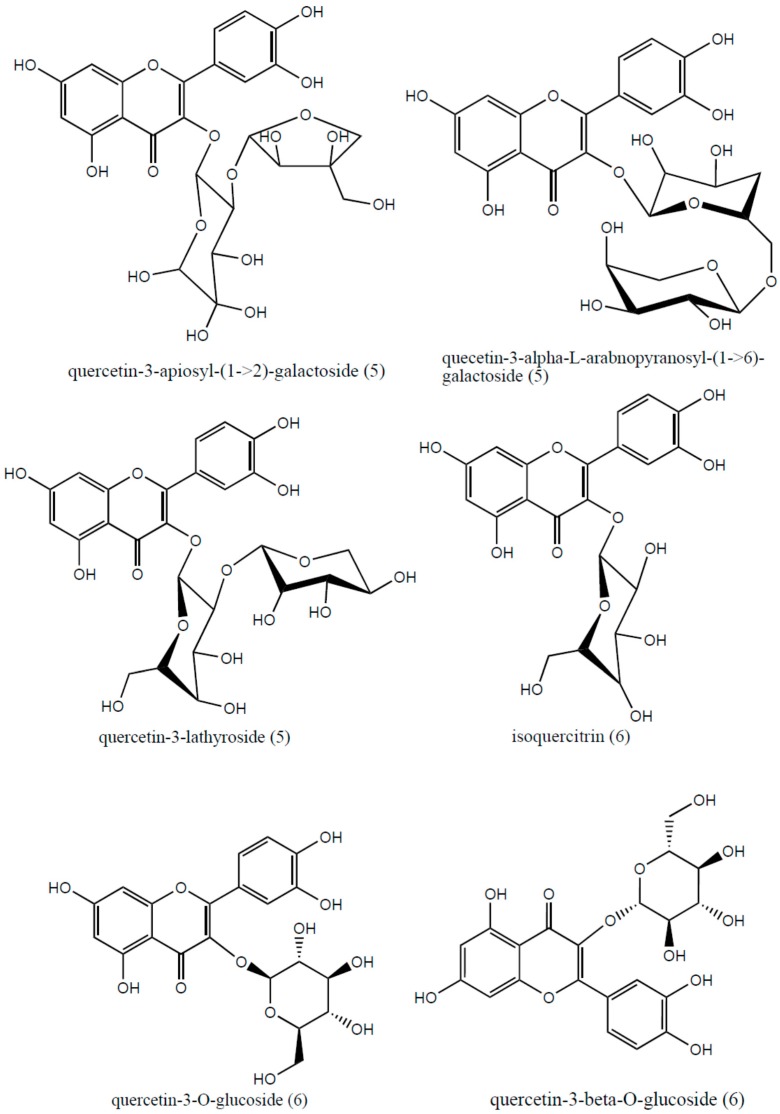
Chemical structure of possible isomers for the compounds **5** and **6** identified by HPLC-ESI-MS in hydroalcoholic extract from leaves of *Himatanthus. drasticus*.

HPLC-ESI-MS analysis allowed the identification of four flavonoids, two iridoids and chlorogenic acid in HDHE (Table 3; Figure 5 and Figure 6). Identification of the compounds was based on the MS fragmentation pattern, literature comparison and the Metlin Scripps Center for Metabolomics database (https://metlin.scripps.edu) [38]. All MS analyzes were performed in positive ion mode. The HPLC-ESI-MS analysis was not able to distinguish some isomers.

Quercetin (**1**) appeared as a peak with *m*/*z* 303.11 [M + H]^+^ compatible with the molecular formula C_15_H_10_O_7_. This compound showed a fragmentation pattern with important peaks at *m*/*z* 285.26, which was attributed to the loss of H_2_O, and at *m*/*z* 199.00 and 149.88 [38]. Plumieride (**2**) was identified based on a peak at *m*/*z* 471.22 [M + H]^+^, compatible with the molecular formula C_21_H_26_O_12_. Fragmentation pattern of this peak revealed a loss of water and a glucose unit at *m*/*z* = 453.28 and *m*/*z* 291.10, respectively [38].

Rutin (**3**) was determined based on the peak *m*/*z* 611.31 [M + H]^+^ compatible with the molecular formula C_27_H_30_O_16_. Fragmentation of this the precursor ion lead to the loss of a rhamnose unit (146 u) and yields an ion at *m*/*z* 465.20, while an aglycone ion at *m/z* 303.08 was obtained through loss of a hexose unit (162 u) [38,39]. Another peak at *m*/*z* 291.12 [M + H]^+^ was compatible with the molecular formula C_15_H_14_O_6_, and was attributed to plumericin or isoplumericin (**4**). Fragmentation provided important peaks at *m*/*z* 263.06 ([M + H]^+^, C_2_H_4_) and *m*/*z* 230.98 ([M^+^], C_2_H_4_O_2_), due to the neutral loss of ethylene unit and an acetyl unit, respectively [38]. However further analysis is required to distinguish which isomer corresponds to the peak.

Also, it was detected a peak at at *m*/*z* 597.27 [M + H]^+^, compatible with the molecular formula C_26_H_28_O_16_. Fragmentation of this peak showed that a successive loss of a pentose and hexose unit, leading to the peaks at *m*/*z* 465.22 and *m*/*z* 303.06, respectively. This compound was identified as quercetin 3-apiosyl-(1->2)-galactoside (**5**) or quercetin 3-α-l-arabinopyranosyl-(1->6)-galactoside (**5**) or quercetin 3-lathyroside (**5**). However, the current study was not able to identify the isomer. The same situation happened with the peak at *m*/*z* 460.20 which was attributed to isoquercitrin or quercetin-3-*O*-glucoside or quercetin-3-β-*O*-glucoside (**6**). A loss a glucose unit (C_6_H_10_O_5_) yielded a fragment at *m*/*z* 303.04, which characterizes an aglycone ion [38]. In addition, a phenolic acid was identified as a peak at *m*/*z* 355.19, which corresponds to the molecular formula C_16_H_18_O_9_. A single fragment at *m*/*z* 162.84 was used for comparison with the Metlin database, allowing to identify this compound as chlorogenic acid (**8**) [38].

## 3. Discussion

This work aimed to analyze some biological activities of HDHE and its fractions obtained from leaves of *H. drasticus*. HDHE showed the best activity against *K. pneumoniae* (bactericidal action (MIC/MBC ratio < 2) [40]. This effect appears to be associated with destabilization of the cell membrane as revealed by PI uptake assay. In fact, the bacterial membrane is the most common target of secondary metabolites, leading to cell lysis, release of cytoplasmic material and consequent cell death [41]. Like other Gram-negative bacteria, *K. pneumoniae* has a double cell wall that constitutes an extra protection against the antimicrobial action by antibiotics, making the search of active compounds against this pathogen a hard task [42].

In the present study, ciprofloxacin associated with the extract presented synergistic and additive actions. Ciprofloxacin is a synthetic quinolone with broad-spectrum antibacterial action. It induces structural damage in bacterial DNA resulting in the activation of the SOS response repair pathway associated to acquisition of virulence and drug resistance genes [43]. The action of *H. drasticus* extract on membrane permeability may explain its ability to increase ciprofloxacin activity, as result of an improvement in drug uptake. In summary, the combination of natural products and antibiotics is considered to have some benefits, such as improvement of therapy effectiveness and drug shelf life [26].

Another important strategy to fight microbial infections is the inhibition of biofilm formation [44]. Biofilm is a major cause of biomaterial implant failure and often limits the life span of various medical devices [16]. Biofilms protect microorganisms against the opsonization, phagocytosis, and epithelial cell removal. Bacterial populations in biofilms are considerably more resistant to antibiotics [45]. HDHE and its fractions had significant inhibitory actions toward *K. pneumoniae* biofilm formation, which, together with synergistic and additive to ciprofloxacin, may represent a potential for drug development.

It was also analyzed whether the active extract had the ability to modulate LPS-induced PBMC proliferation. LPS (the main component of the cell wall of Gram-negative bacteria) is a potent inducer of human T-lymphocyte proliferation as part of host pro-inflammatory response [46]. In addition, the anti-inflammatory properties of some phytochemicals present in HDHE have been reported [47,48,49,50]. The use of anti-inflammatory agents is of great importance in the treatment of microbial infection [51,52]. Considering frequent empirical use of this species in popular medicine, it is also extremely important to evaluate the toxicity of the plant. In the two tests performed with HDHE of *H. drasticus*, no toxicity was observed. These results suggest a selective action of this extract towards *K. pneumoniae* membrane.

Finally, the presence of several bioactive compounds was detected by HPLC-MS, which may explain the pharmacological characteristics of the plant under study. For example, plumieride, plumericin, and isoplumericin have proven antimicrobial, anti-inflammatory and antiproliferative activities [53,54,55]. Other compounds detected in HDHE have been also reported as promising alternative to treat antimicrobial infections and inflammatory disorders such as quercetin and chlorogenic acid [56,57,58,59,60,61].

## 4. Material and Methods

### 4.1. Botanical Material

Leaves of *H. drasticus* were collected in the urban area of São Luís-MA (2°30′48″ S 44°17′20.7″ W), in November/2015. A sample of the plant (branch with leaf, flower and fruit) was sent to the Herbarium “*Ático Seabra*” of the Federal University of Maranhão (UFMA) for identification and was cataloged under registration number 1032/2015.

### 4.2. Preparation of Hydroalcoholic Extract and Its Fractions

After harvesting, leaves (500 g) were cleaned with tap water and placed in an amber bottle containing a 70% ethanol solution, at a ratio of 1:3 *m*/*v*. The macerate underwent a daily shaking process (three times per day) for 48 h. After maceration, the solution was filtrated and concentrated in a rotary evaporator under reduced pressure at 50 °C. After concentration, the final solution was termed *H. drasticus* hydroalcoholic extract (HDHE). HDHE was fractionated by liquid-liquid partition using 10 g of dry extract, which was resuspended in a hydromethanolic solution (1:9 water:methanol, *v*/*v*). This solution was submitted to successively liquid-liquid partitions using solvents with increasing polarity, yielding a hexane fraction (HDHEF), ethyl acetate fraction (HDEAF) and butanol fraction (HDBF).

### 4.3. Antimicrobial Assays

#### 4.3.1. Test Microorganisms

The microbial strains used in this work were obtained from the Culture Collection (Laboratory of Microbiology) of the CEUMA University, including *Staphylococcus aureus* (ATCC 25923), *Pseudomonas aeruginosa* (ATCC 27853), *Salmonella enterica* serovar typhimurium (ATCC 14028), *Listeria monocytogenes* (ATCC 15313), *Escherichia coli* (ATCC 25922), *Klebsiella pneumoniae* (ATCC 10031), *Acinetobacter baumanni* (ATCC 19606), *Enterococcus faecalis* (ATCC 19433).

#### 4.3.2. Antimicrobial Agar Diffusion Assay

From recent overnight cultures, microbial suspensions with turbidity equivalent to 0.5 tube of the McFarland scale (1.5 × 10^8^ CFU/mL) were prepared. Each bacterial strain was seeded on Mueller-Hilton agar (MHA) plates (DIFCO-Becton Dickinson Microbiology Systems, Bergen, NJ, USA). Next, wells (6 mm diameter) prepared in MHA plates were filled with 50 μL of 200,000 µg/mL extract/fractions solutions (resulting in 10,000 µg of each sample in each well). Dimethylsufoxide (DMSO; 50 μL of a 1% solution; Sigma-Aldrich^®^, St. Louis, MO, USA) and ciprofloxacin (50 μg per well; Laborclin, Paraná, Brazil) were used as negative and positive controls, respectively. The plates were incubated at 35 °C. After 24 h, the formation of inhibition diameter zone (IDZ) around the wells was recorded (CLSI, 2015). The experiments were performed in triplicate.

#### 4.3.3. Minimum Inhibitory Concentration (MIC) and Minimum Bactericidal Concentration (MBC)

HDHE and its fractions were serially diluted in 96-wells plates containing Mueller-Hinton broth (DIFCO-Becton Dickinson Microbiology Systems, Bergen, NJ, USA). Concentrations ranging from 50,000 µg/mL to 48.82 µg/mL were tested. Subsequently, 1 μL of a microorganism suspension (1.5 × 10^8^ CFU/mL) was added in each well. Ciprofloxacin (50 µg/mL suspension) and DMSO (1% suspension) were used as controls. The plates were incubated at 35 °C for 24 h, prior the addition of 30 μL of 0.03% resazurin sodium solution (Sigma-Aldrich^®^), an oxide-reducing indicator). After 40 min, changes from blue color to pink color were considered as microbial growth. Minimum Inhibitory Concentration (MIC) was defined as the lowest concentration capable of inhibiting bacterial growth. For determination of Minimum Bactericidal Concentration (MBC), samples (10 μL) of those wells where the inhibition was observed were transferred to MHA plates. After 24 h incubation, MBC was defined as the lowest concentration able of eliminate bacteria.

#### 4.3.4. Biofilm Assay

For biofilm formation, 10 μL of *K. pneumoniae* bacterial solution (prepared as described in MIC section) were mixed to 140 μL of MHB and 50 μL of extract and fractions to reach subinhibitory concentrations (780 µg/mL and 1560 µg/mL). After 24 h of incubation at 37 °C, the formed biofilm was fixed with methanol (P.A.), stained with violet crystal (0.1%) and washed with ethanol (P.A.). As negative control, culture medium with bacterial solution was used. Biofilm biomass was measured using a spectrophotometer at 550 nm (based on [62]).

#### 4.3.5. Combined Effects of HDHE and Ciprofloxacin

The effect of the active extract in combination with antibiotics was evaluated against *K. pneumoniae* ATCC 10031 by agar diffusion assay and microdilution assay. The diffusion agar assay was performed as described above, except for the addition of hydroalcoholic extract (500 µg, 1000 µg or 5000 µg) to ciprofloxacin disks (5 μg). After 24 h, each IDZ was read and compared with those obtained using ciprofloxacin alone. In the microdilution assay, different combinations of HDHE and ciprofloxacin were tested as described in the MIC section. The results were used to calculate the fractional inhibitory concentrations (FIC), using the following equation [17]:
$\mathrm{FIC}:\frac{\mathrm{MICA}}{\mathrm{MICextract}}+\frac{\mathrm{MICB}}{\mathrm{MICdrug}}=$FIC ≤ 0.5: synergistic effect0.5 < FIC ≤ 1: additive effect1.0 < FIC ≤ 4: no interactionFIC > 4: antagonism

MMICA: MIC of extract when in combination with drug; MICextract: MIC of extract alone; MICB: MIC of drug when in combination with extract; MICdrug:MIC of drug alone.

#### 4.3.6. Evaluation of Membrane Integrity

Aliquots of 1 mL of *K. pneumoniae* suspension (1.5 × 10^8^ CFU/mL) were treated with different concentrations of HDHE (0.5×, 1×, 2×, 4× the MIC) for 4 h. Microorganisms killed by heat (100 °C for 10 min) were used as positive control, and bacteria grown without extract were used as negative control. Bacteria were centrifuged at 3000 RPM for 10 min, washed (2 times) and resuspended in 30 μM propidium iodide (PI; eBioscience, ThermoFisher, Waltham, MA, USA). The tubes were incubated for 10 min in the dark. The samples were collected on the flow cytometer (BD Accuri™ C6, 20,000 events per sample) and data were analyzed using the FlowJo software (TreeStar, Ashland, OR, USA). The results represent the percentage of incorporation of PI by bacteria (loss of membrane integrity).

### 4.4. Assays Using Human Blood

Blood samples were collected from three healthy volunteers with no recent history of taking either antibiotic or anti-inflammatory drugs, and/or infectious or inflammatory diseases in the last 3 weeks prior to sample collection; after a written informed consent was obtained. The study was reviewed and approved by the Human Research Ethics Committee of the *Universidade CEUMA* (CEP-UNICEUMA) under registration number no. 44785215.6.0000.5084. The tests were performed in accordance with the Declaration of Helsinki 1975, revised in 2008.

#### 4.4.1. Hemolysis Assay

After collection by venipuncture, whole human blood samples (5 mL) were centrifuged for 10 min at 1500 RPM, serum was discarded and pellet was washed 3 times and resuspended in PBS (pH = 7.4), yielding a 1% erythrocytes suspension. The erythrocytes suspensions were treated with HDHE (25,000 µg/mL, 12,500 µg/mL, 6250 µg /mL, 3125 µg/mL, 1560 µg/mL), PBS (negative control), or hydrogen peroxide (1 mM, positive control). After shaking for 60 min, the samples were centrifuged and the absorbance of the supernatant was read at 540 nm (Full-Citomatic Microplate Reader MB-580, Heales, Shenzhen, Guangdong, China).

#### 4.4.2. Cell Proliferation Assay with PBMC

Samples of 5 mL of human peripheral blood were added to histopaque–containing tube (1:1 ratio). After centrifugation, peripheral blood mononuclear cells (PBMC) were collected from the formed ring. Cells were washed with PBS and resuspended in Dulbecco’s Modified Eagle Medium (DMEM, Gibco^®^ by Life Technology™, Grand Island, NY, USA) culture medium supplemented with 10% fetal bovine serum (FBS) and 1% of antibiotics (streptomycin, penicillin) and 1% of antifungal (amphotericin B; Sigma-Aldrich^®^).

Samples of 1 mL of PBMC (0.6 × 10^6^ cells per tube) were labeled with 10 μg/mL carboxyfluorescein succinimidyl ester (CFSE; eBioscience) for 30 min at 37 °C, washed (2 times) and treated with the extract at the concentrations of 780 µg/mL and 1560 µg /mL in the presence or absence of LPS (100 μg/mL of *E. coli*-derived lipopolysaccharide; Sigma-Aldrich). PBMC treated with LPS or cultured with culture medium alone were used as controls. After 72 h, cells were harvested and proliferation was assessed by flow cytometry (BD Accuri™ C6, 20,000 events per sample) and data were analyzed using the FlowJo software. The results represent the percentage of cells that underwent cell division.

### 4.5. Chemical Characterization of HDHE

The HPCL-ESI-MS analyses were performed at the Central Instrumentation Laboratory of the University of São Paulo using an LC-20A chromatography system (Shimadzu, Kyoto, Japan) comprised of a CBM-20A controller, LC-20AD pump, SPD-20AC detector and a CTO-20A column oven and SIL 20AC—Shimadzu detector, coupled to an Amazon Speed ETD ion trap spectrometer (Bruker, Bremen, Germany). The HPLC analysis was performed using a Luna C18 (250 × 4.6 mm–5 µm) column (Phenomenex, Torrance, CA, USA) held at 40 °C. The following elution gradient was employed with a flow rate of 1 mL·min^−1^: solvent A = aqueous formic acid, 0.1 % (*v*/*v*); solvent B = MeCN. Elution profile = 0–5 min, isocratic 5%B; 5–6 min, linear gradient 5–20%B; 6–10 min, isocratic 20%B; 10–15 min, linear gradient 20–50%B; 15–20 min, linear gradient 50–72%B; 20–30 min, isocratic 72%B; 30–40 min, linear gradient 72–100%B; 40–45 min, linear gradient 100–5%B, 45–50 min, isocratic 5%B. The gradienty profile is shown in the Supplemental Material. UV chromatograms were recorded at 254 nm and 280 nm. HPLC-MS TIC chromatograms were recorded between *m*/*z* 100 and *m*/*z* 1500 in positive ion mode (ESI+). The following mass spectrometry parameters were maintained in the analysis: capillary 4500 V; drying gas flow 7.00 L·min^−1^; drying gas temperature, 300 °C; nebulizer gas pressure, 27.00 psi. After HPLC-ESI-MS run, the retention time and *m*/*z* for each chromatographic peak was used to determine MS/MS fragmentation parameters.

### 4.6. Statistical Analysis

Statistical analyses were performed using the software GraphPad Prism version 5.01 (GraphPad Software Inc., La Jolla, CA, USA). Data were analyzed by two-way analysis of variance (ANOVA) followed by Tukey test. A *p*-value of < 0.05 was considered as statistically significant. All tests were performed in at least two independent assays in quadruplicate. For flow cytometry assays, at least 20,000 cells were analyzed.

## 5. Conclusions

The results showed that HDHE can act as antimicrobial against *K. penumoniae*, since significantly diminished its viability and inhibited biofilm formation. The action mechanism is associated to the damage of bacterial membrane, which may be also related to HDHE ability to increase the activity of ciprofloxacin, as we demonstrated synergistic and additive actions. HDHE also reduces proliferation of human PBMCs stimulated with LPS, presenting no significant cytotoxicity. The bioactive compounds found in the extract by HPLC-MS analysis (plumieride, plumericin, and isoplumericin, quercetin and chlorogenic acid) have been reported as antimicrobial and anti-inflammatory agents. Particularly, the results regarding the inhibition of *K. pneumoniae* biofilm encourages further studies with *H. drasticus* derivative products to develop new compounds to be used in control of device-related infections.

## Figures and Tables

**Table 1:** Antimicrobial activity of hydroalcoholic extract from leaves of *Himatanthus drasticus* and its fractions.

Bacterial Species	HDHE	HDHF	HDEAF	HDBF	Ciprofloxacin
	Inhibition Diameter Zone (mm)				
*Staphylococcus aureus*	-	-	-	10 ± 0.5	33 ± 0.5
*Pseudomonas aeruginosa*	-	20 ± 0.5	-	25 ± 0.5	43 ± 0.0
*Salmonella enterica*	-	-	-	-	35 ± 0.5
*Listeria monocytogenes*	12 ± 0.5	-	30 ± 0	-	36 ± 0.5
*Escherichia coli*	-	-	-	-	45 ± 0.5
*Klebsiella. pneumoniae*	16 ± 0.5	-	-	-	40 ± 1.1
*Acinetobacter baumannii*	-	-	-	-	40 ± 0.0
*Enterococcus faecalis*	-	-	-	-	28 ± 1.1

HDHE: *H. drasticus* hydroalcoholic extract; HDHF: *H. drasticus* hexane fraction; HDEAF: *H. drasticus* ethyl acetate fraction; HDBF: *H. drasticus* butanolic fraction. (-) No activity. The tests were performed in two independent assays in quadruplicate.

**Table 2:** Minimum Inhibitory (MIC) and Minimum Bactericidal Concentrations (MBC) of hydroalcoholic extract from leaves of *Himatanthus. drasticus* and its fractions.

Bacterial Species	Header	HDHE	HDHF	HDEAF	HDBF	Ciprofloxacin
*Staphylococcus aureus*	**MIC:**	NE	NE	NE	>50,000	0.78
	**MBC:**	NE	NE	NE	>50,000	0.78
*Pseudomonas aeruginosa*	**MIC:**	NE	>50	NE	>50,000	25
	**MBC:**	NE	>50	NE	>50,000	25
*Listeria monocytogenes*	**MIC:**	2500	NE	>50,000	NE	1.56
	**MBC:**	2500	NE	>50,000	NE	1.56
*Klebsiella pneumoniae*	**MIC:**	6250	NE	NE	12,500	12.5
	**MBC:**	6250	NE	NE	12,500	12.5

HDHE: *H. drasticus* hydroalcoholic extract; HDHF: *H. drasticus* hexane fraction; HDEAF: *H. drasticus* ethyl acetate fraction; HDBF: *H. drasticus* butanolic fraction. NE: not evaluated. Concentrations ranging from 50,000 µg/mL to 48.82 µg/mL were tested for each plant sample. For ciprofloxacin, concentrations ranging from 50 µg/mL to 0.048 µg/mL were tested. The tests were performed in two independent assays in quadruplicate.

**Table 3:** Identification of compounds in the hydroalcoholic extract from leaves of *Himatanthus. drasticus* by HPLC-ESI-MS analysis.

Compound	Rt (min)	*m/z* [M + H]^+^	λ_MAX_ (nm)	MS^2^ Main Fragments
Chlorogenic acid	11.6	355.19 ([M + H]^+^)	254	162.84
Plumericin or isomers	13.0	291.12 ([M + H]^+^)	254, 280	263.06, 230.98
Quercetin	13.5	303.11 ([M + H]^+^)	254, 280	285.26
Quercetin 3-lathyroside or isomers	13.5	597.27 ([M + H]^+^)	254, 280	465.22, 303.06
Rutin	14.8	611.31 ([M + H]^+^)	254, 280	465.20, 303.08
Isoquercetrin or isomers	15.8	465.20 ([M + H]^+^)	254, 280	303.04
Plumieride	24.4	493.41 ([M + Na]^+^)	254, 280	448.41

## Supplementary Materials

The supplementary materials are available online.
