# Supplementary material for: Himatanthus drasticus Leaves: Chemical Characterization and Evaluation of Their Antimicrobial, Antibiofilm, Antiproliferative Activities
Source: Molecules. 2017 May 31;22(6):910. doi: 10.3390/molecules22060910 (PMC6152732; doi:10.3390/molecules22060910)

Supplementary material

Mass spectrometry:

Mass spectrometer**:** Amazon Speed ETD - Bruker

Capillary: 4500V

Nebulizer: 27.00 psi

Dry Gas: 7.00 L.min^{-1}

Dry Temp: 300 °C

Scan begin: 100 *m/z*

Scan end: 1500 *m/z*

ESI+

HPLC:

Controler: CBM-20A- Shimadzu

Pump: LC-20AD Shimadzu

Detector: SPD-20A - Shimadzu

Oven: CTO-20A - Shimadzu

Autoinjector: SIL 20AC – Shimadzu

Solvent A: Aqueous formic acid 0,1%

Solvente B: MeCN

Column: Phenomenex – Luna C18 (250 x4.6mm – 5um)

Oven temperature: 40°C

Flow rate: 1 ml/min

Method: 0-5 min, isocratic 5%B; 5-6 min, linear gradient 5-20%B; 6-10 min, isocratic 20%B; 10-15 min, linear gradient 20-50%B; 15-20 min, linear gradient 50-72%B; 20-30min, isocratic 72%B; 30-40 min, linear gradient 72-100%B; 40-45 min, linear gradient 100-5%B, 45-50 min, isocratic 5%B.

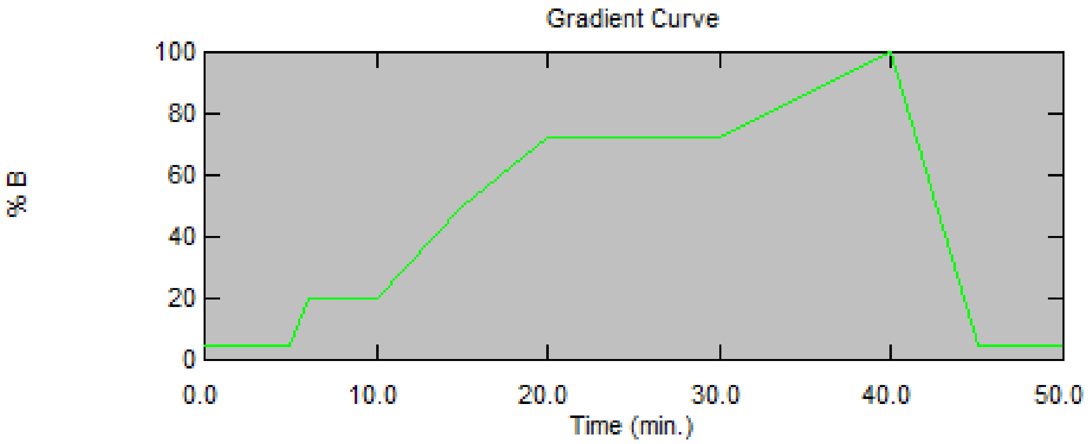

UV Chromatogram of the hydroalcoholic extract (HDHE) at 254 nm.

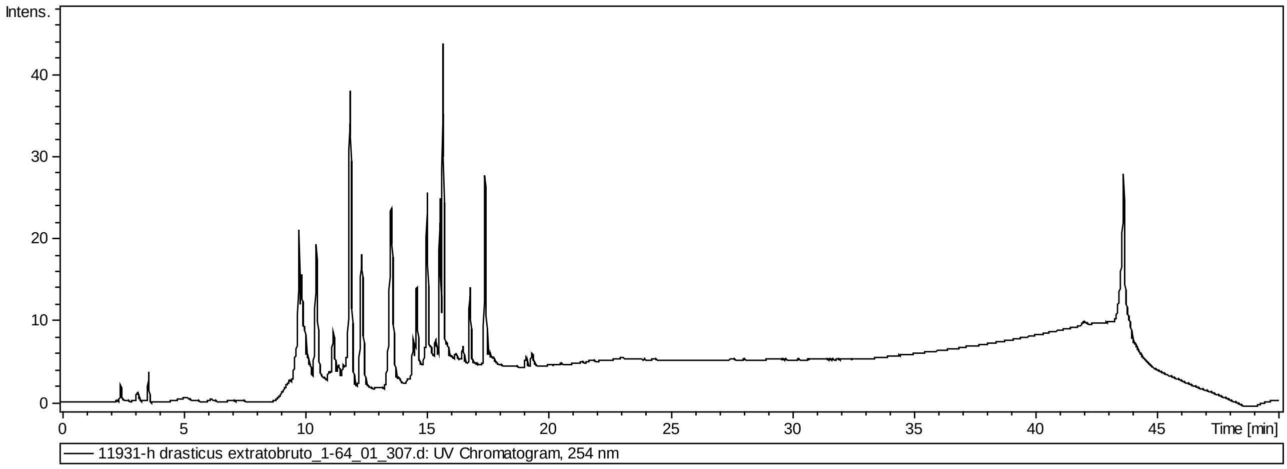

UV Chromatogram of the hydroalcoholic extract (HDHE) at 280 nm

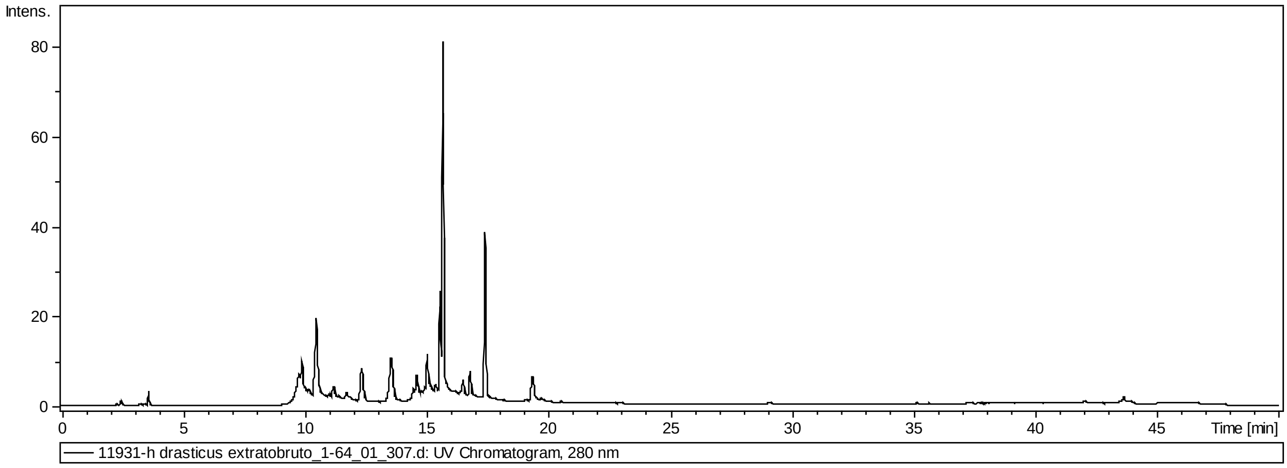

Ion Chromatogram of the hydroalcoholic extract (HDED)

ESI+

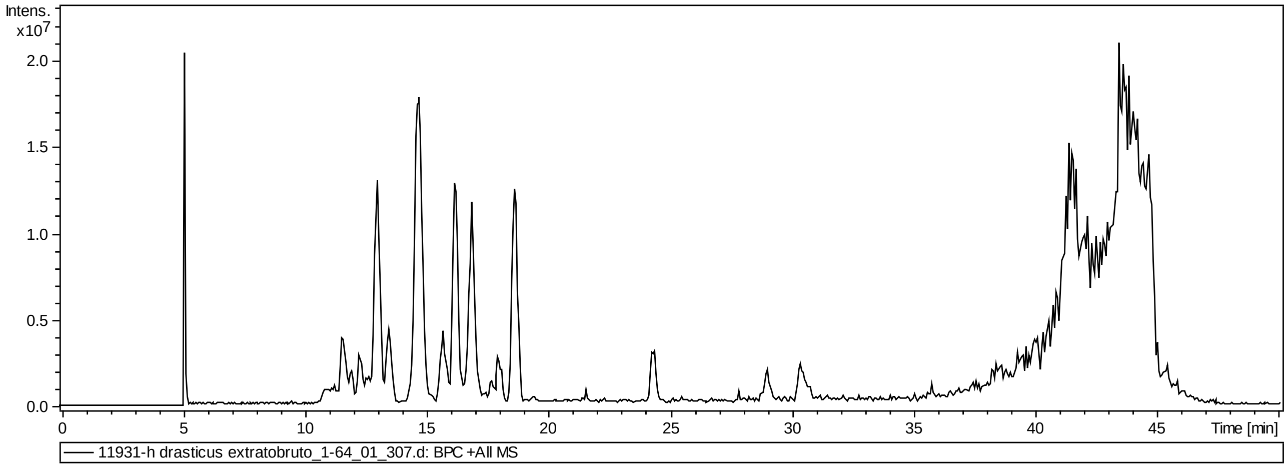

(+)-ESI-IT-MS spectra of quercetin (1) *m/z* 303.11 [M+H]+

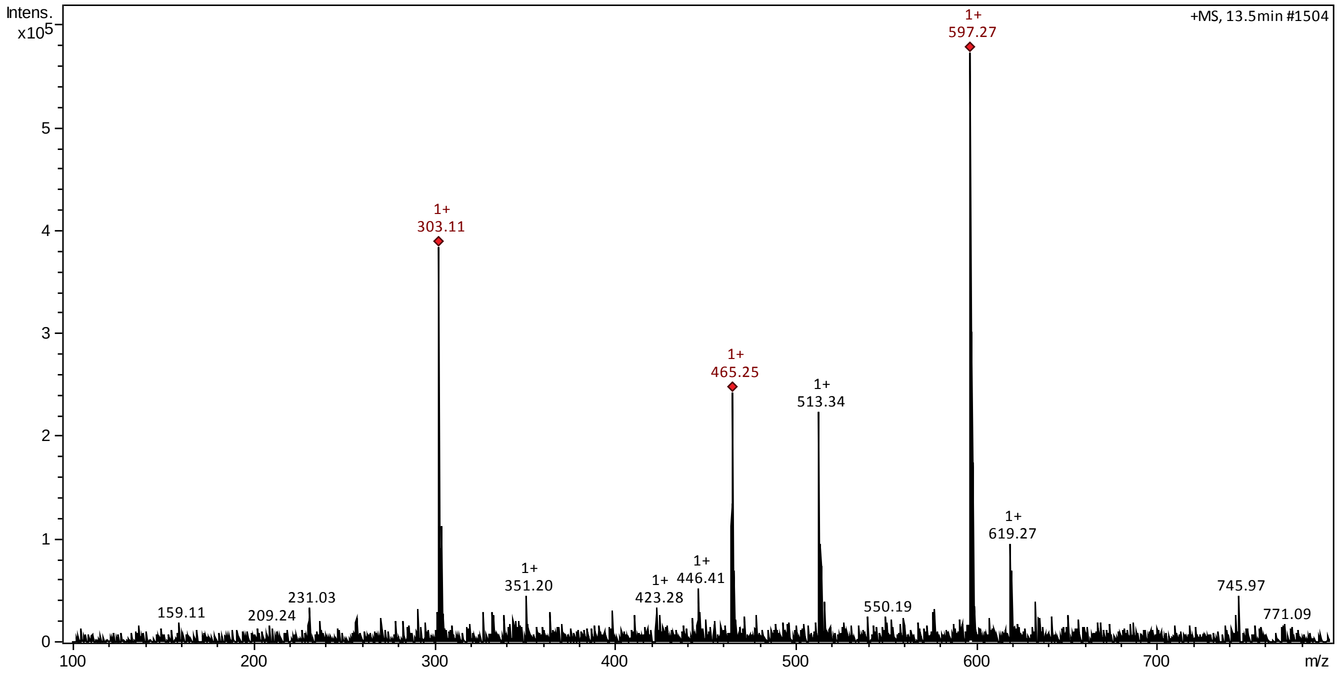

(+)-ESI-IT-MS/MS spectra of quercetin (1) *m/z* 303.11 [M+H]+

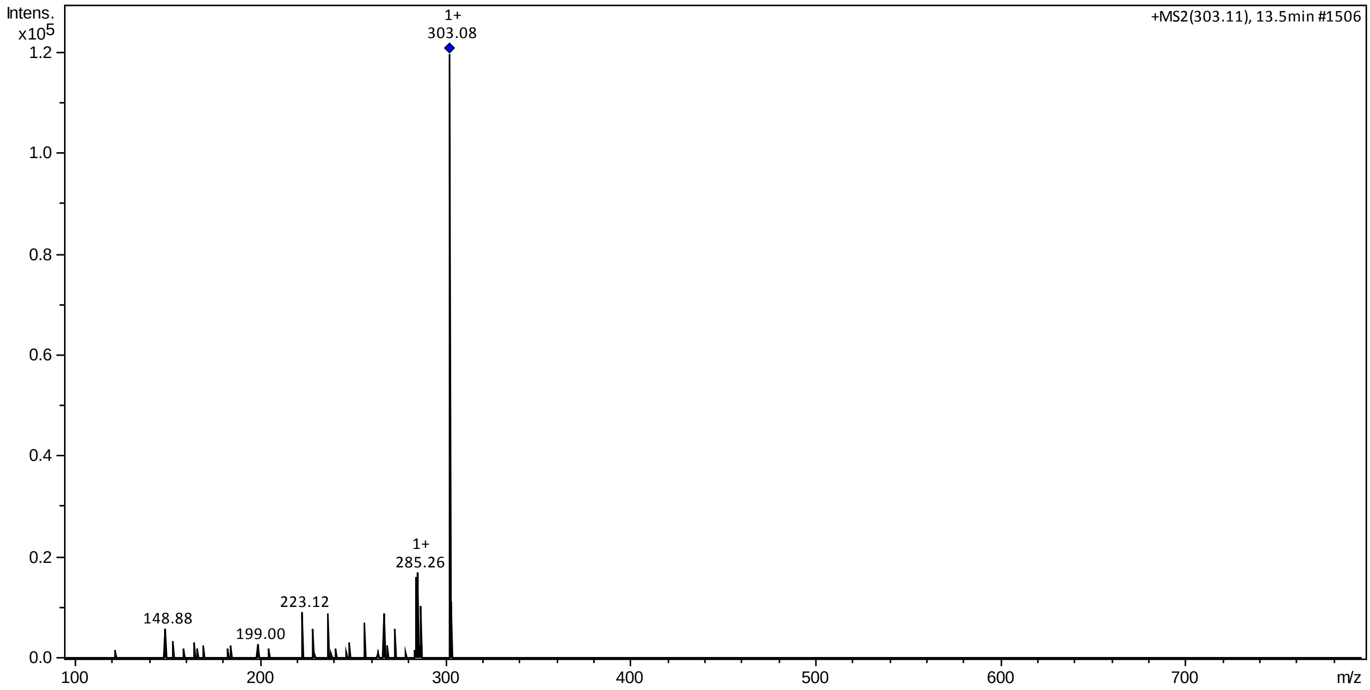

(+)-ESI-IT-MS spectra of plumieride (2) m/z 493.41 $[M+Na]^+$

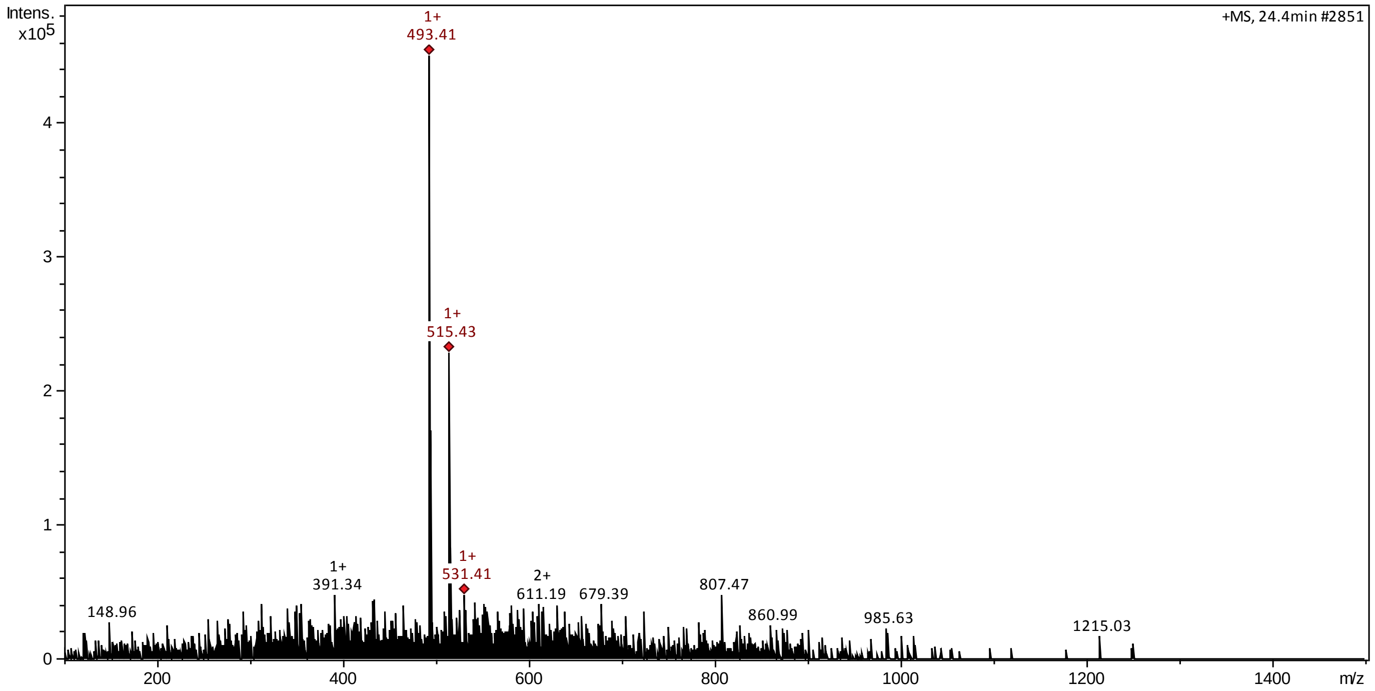

(+)-ESI-IT-MS/MS spectra of plumieride (2) m/z 493.41 $[M+Na]^+$

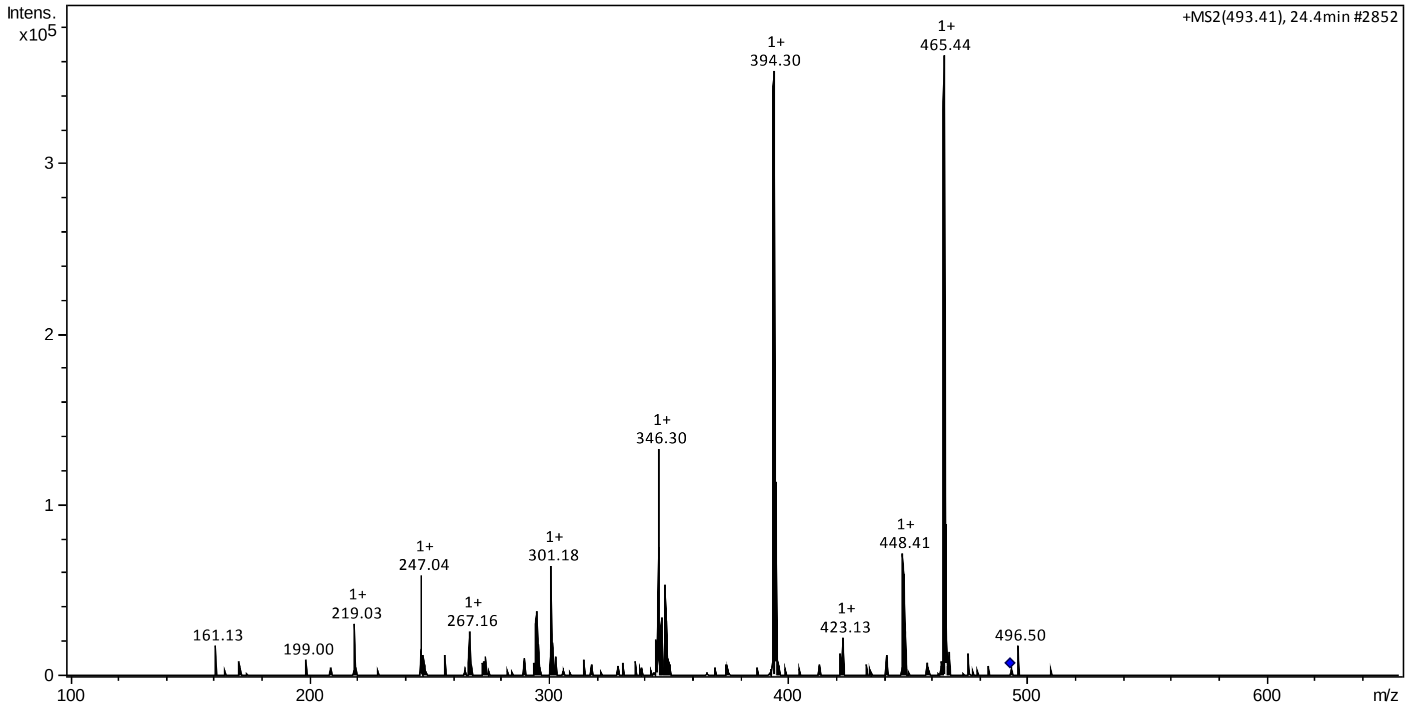

(+)-ESI-IT-MS spectra of rutin (3) m/z 611.31 $[M+H]^+$

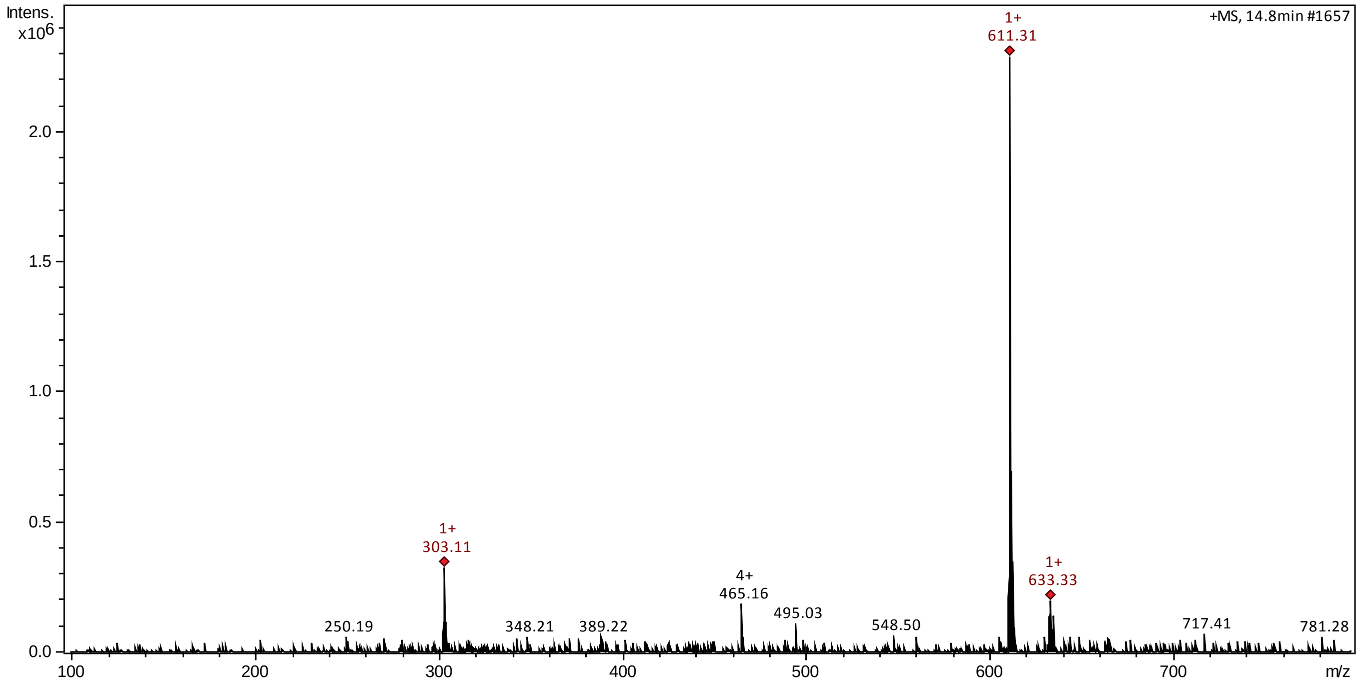

(+)-ESI-IT-MS/MS spectra of rutin (3) *m/z* 611.31 $[M+H]^+$

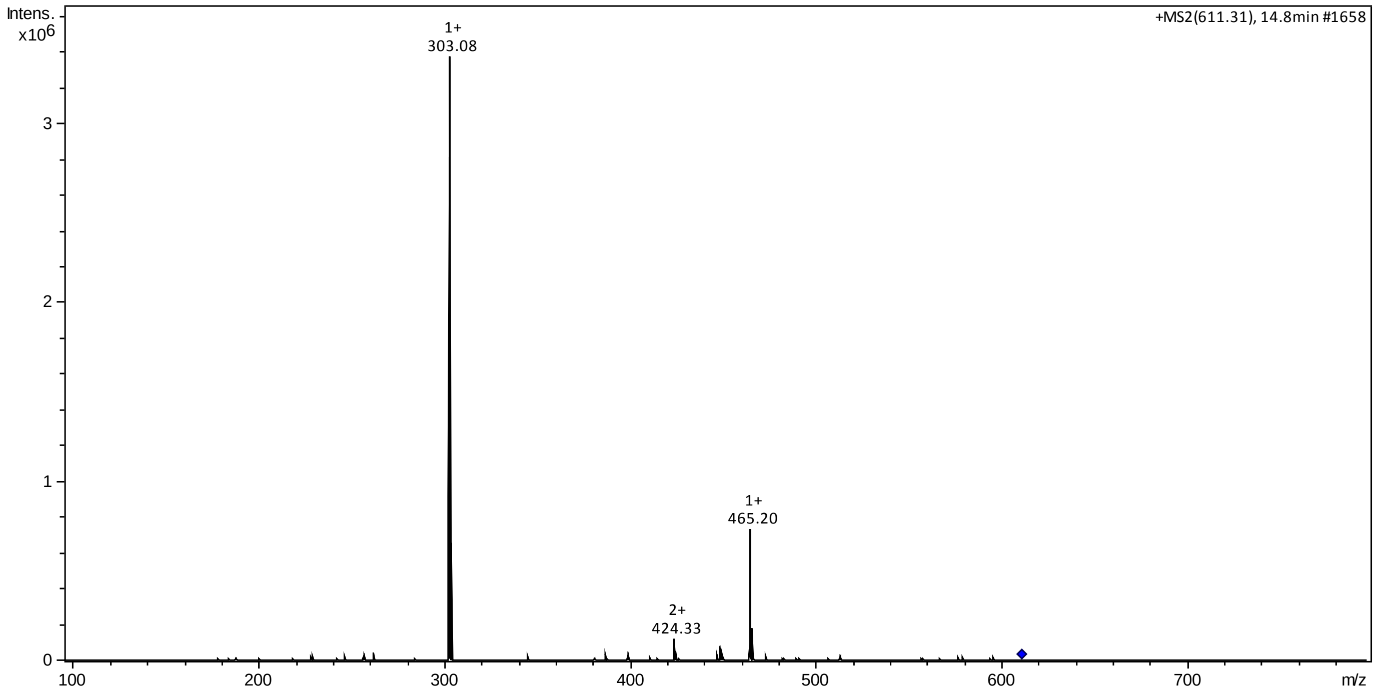

(+)-ESI-IT-MS spectra of plumerin (4) or isoplumericin (4) *m/z* 291.12 [M+H]+.

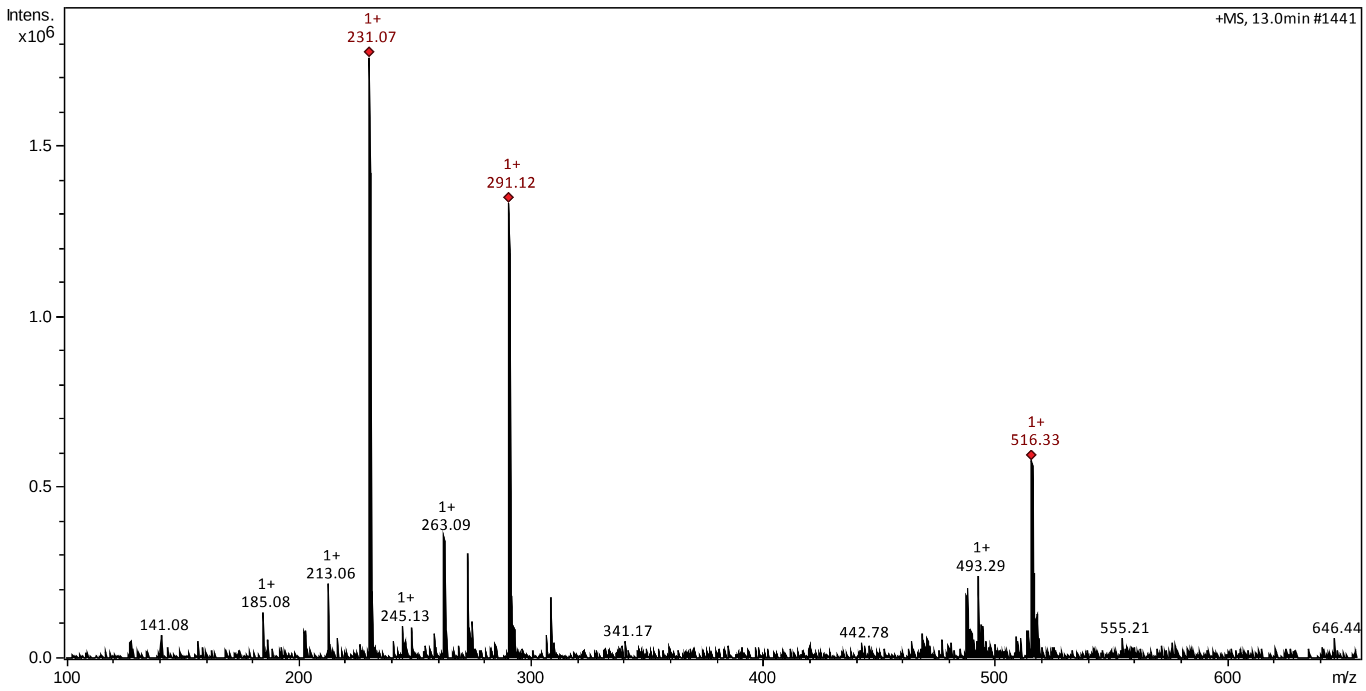

(+)-ESI-IT-MS/MS spectra of plumerin (4) or isoplumericin (4) *m/z* 291.12 [M+H]+.

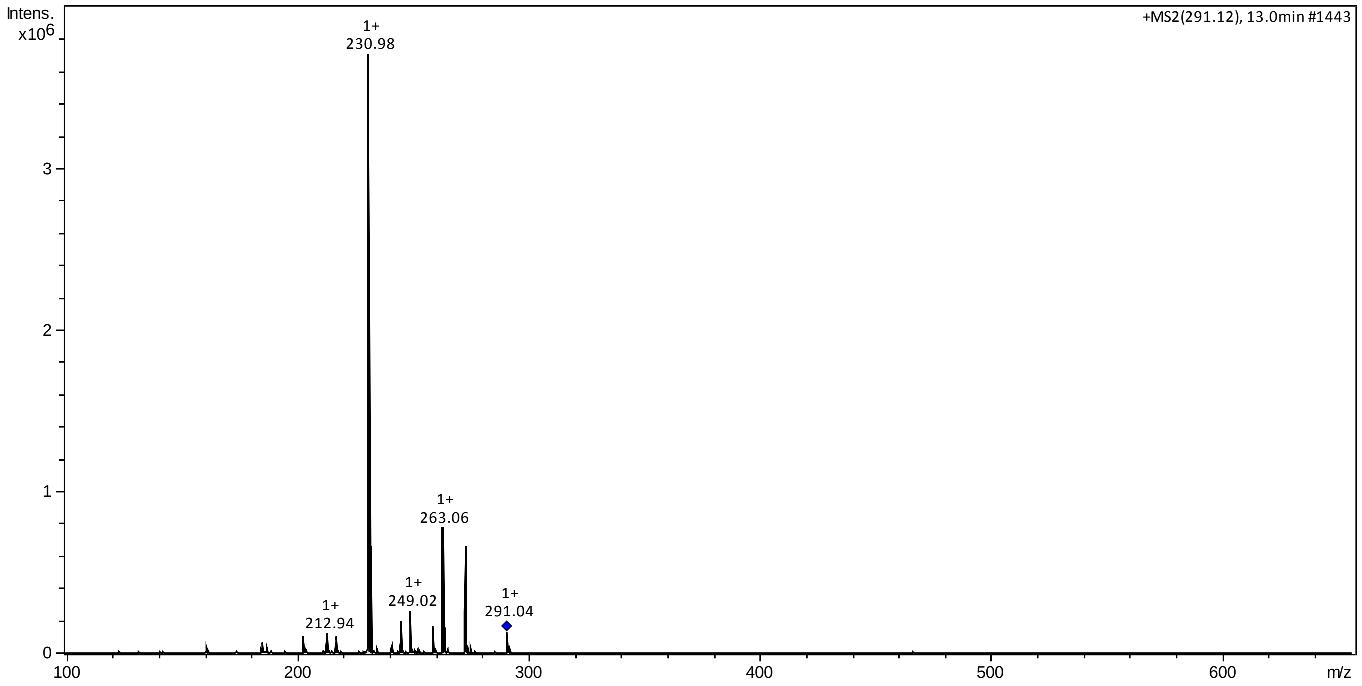

(+)-ESI-IT-MS/MS spectra of quercetin 3-lathyroside (5) or isomers *m/z* 597.27 [M+H]$^+$

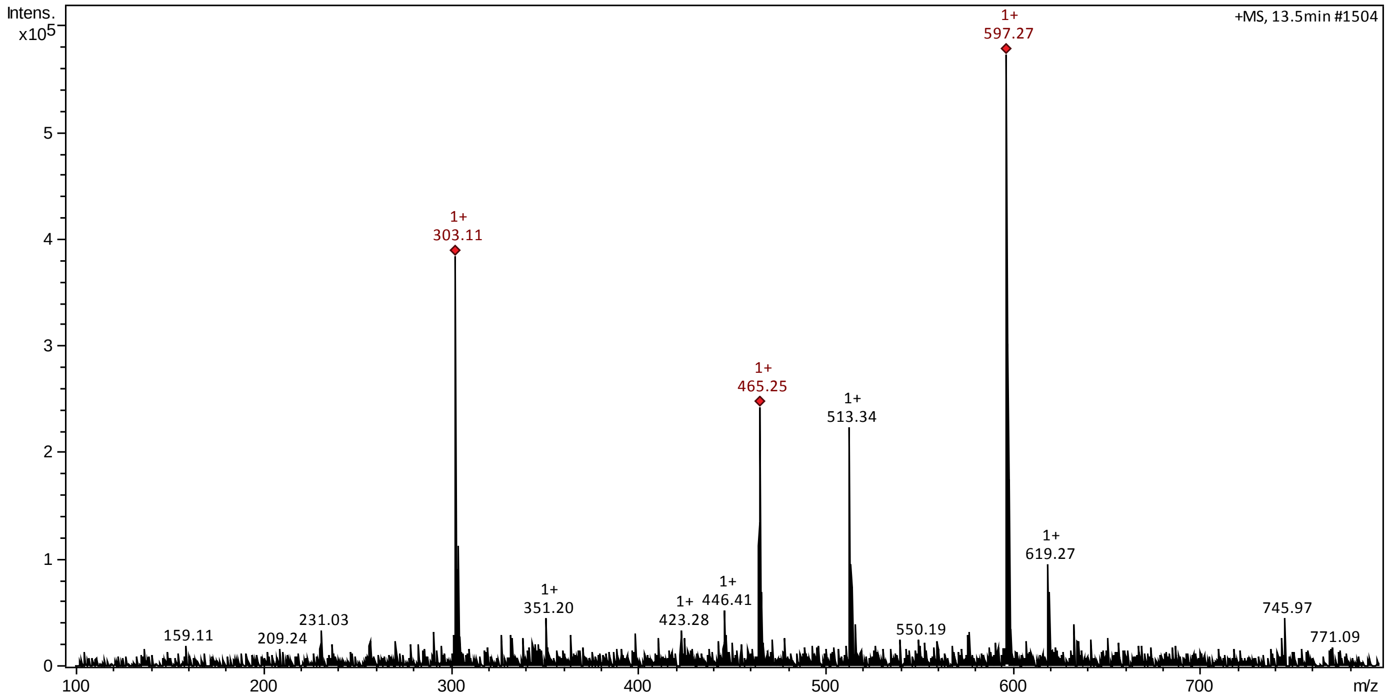

(+)-ESI-IT-MS/MS spectra of quercetin 3-lathyroside (5) or isomers *m/z* 597.27 [M+H]$^+$

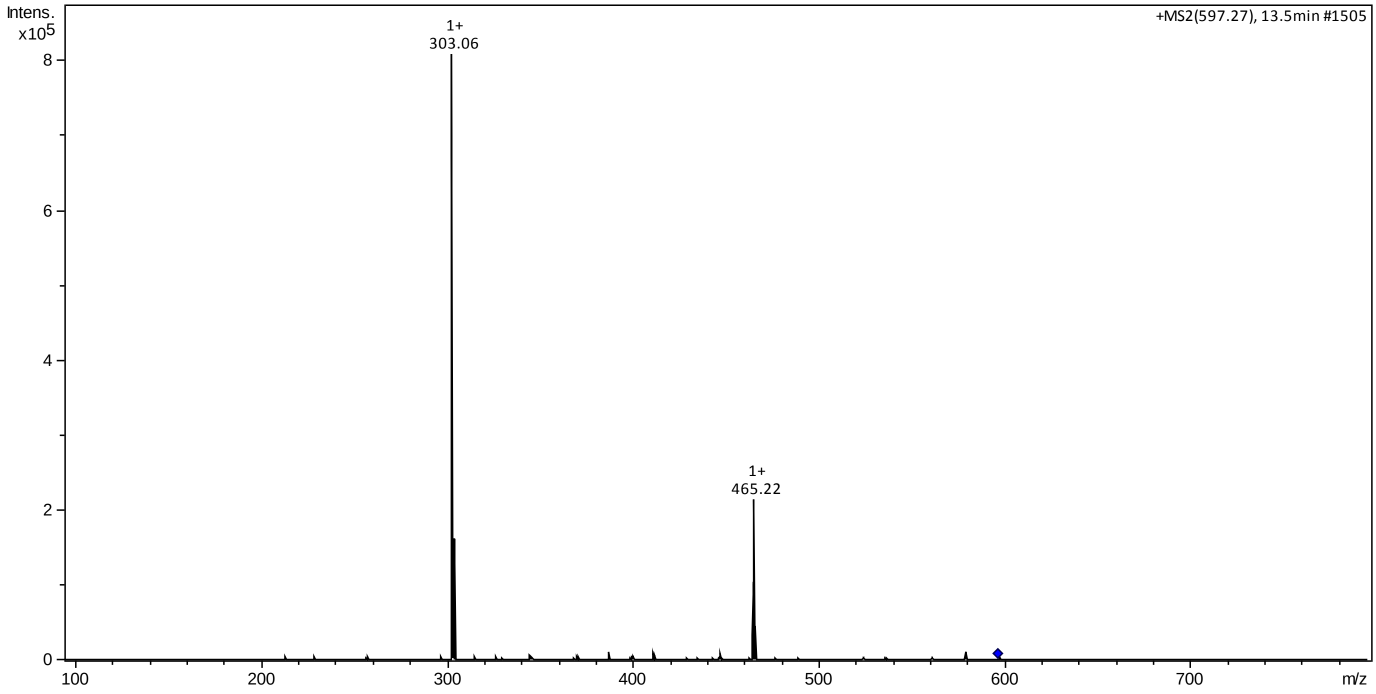

(+)-ESI-IT-MS spectra of isoquercitrin (6) or isomers *m/z* 465.20 [M+H]$^+$

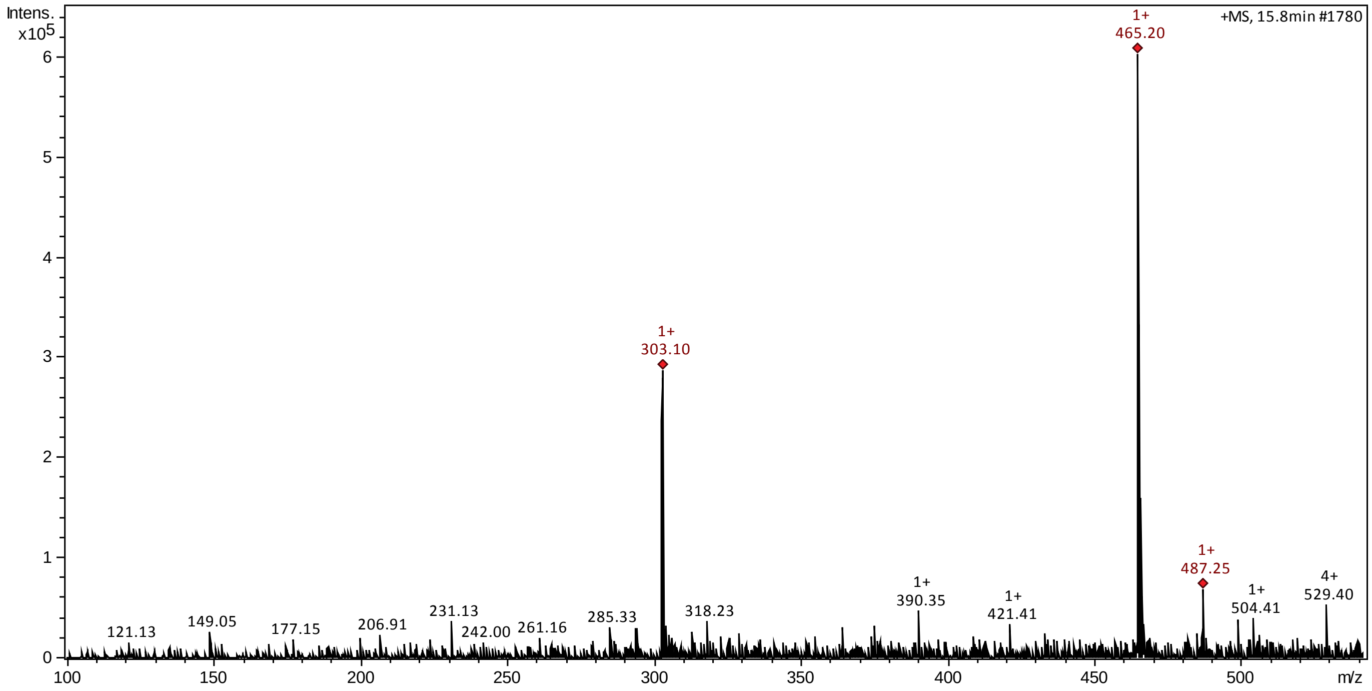

(+)-ESI-IT-MS/MS spectra of isoquercitrin (6) or isomers *m/z* 465.20 [M+H]$^+$

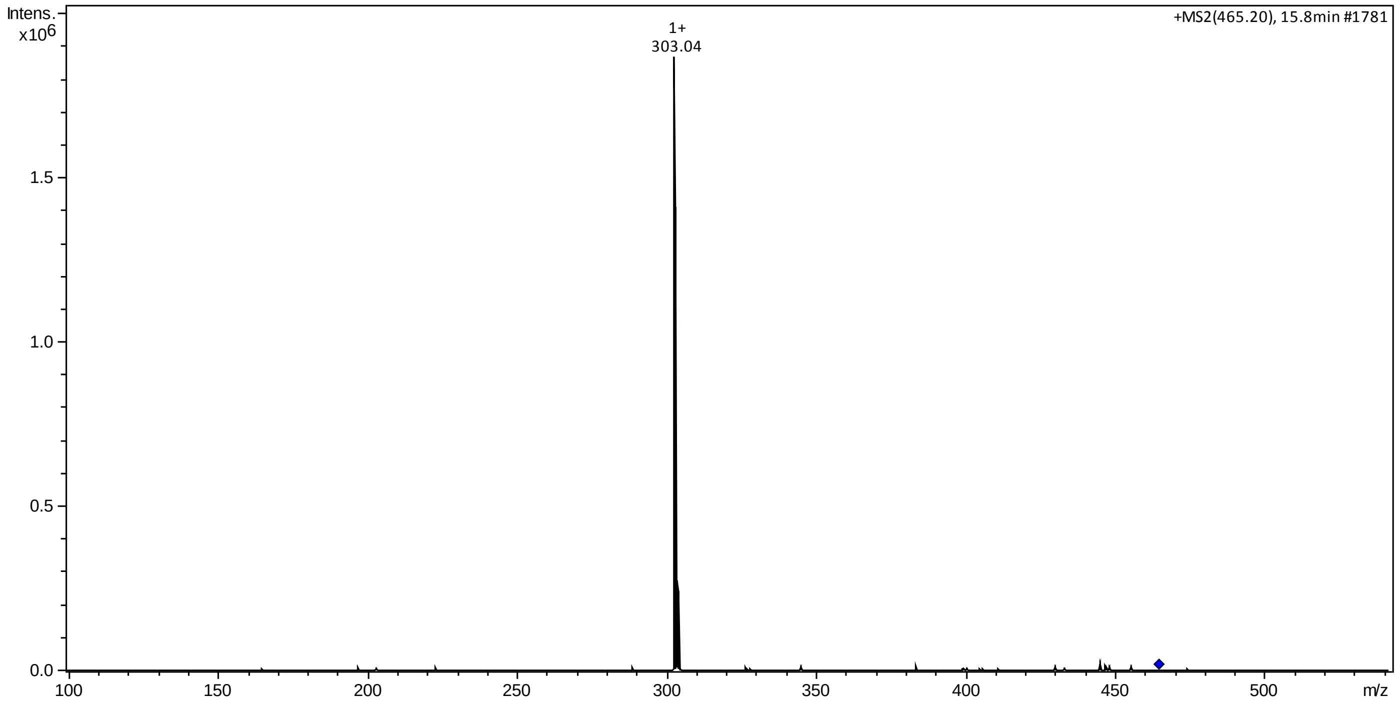

(+)-ESI-IT-MS spectra of chrologenic acid (7) *m*/*z* 355.19 [M+H]$^+$

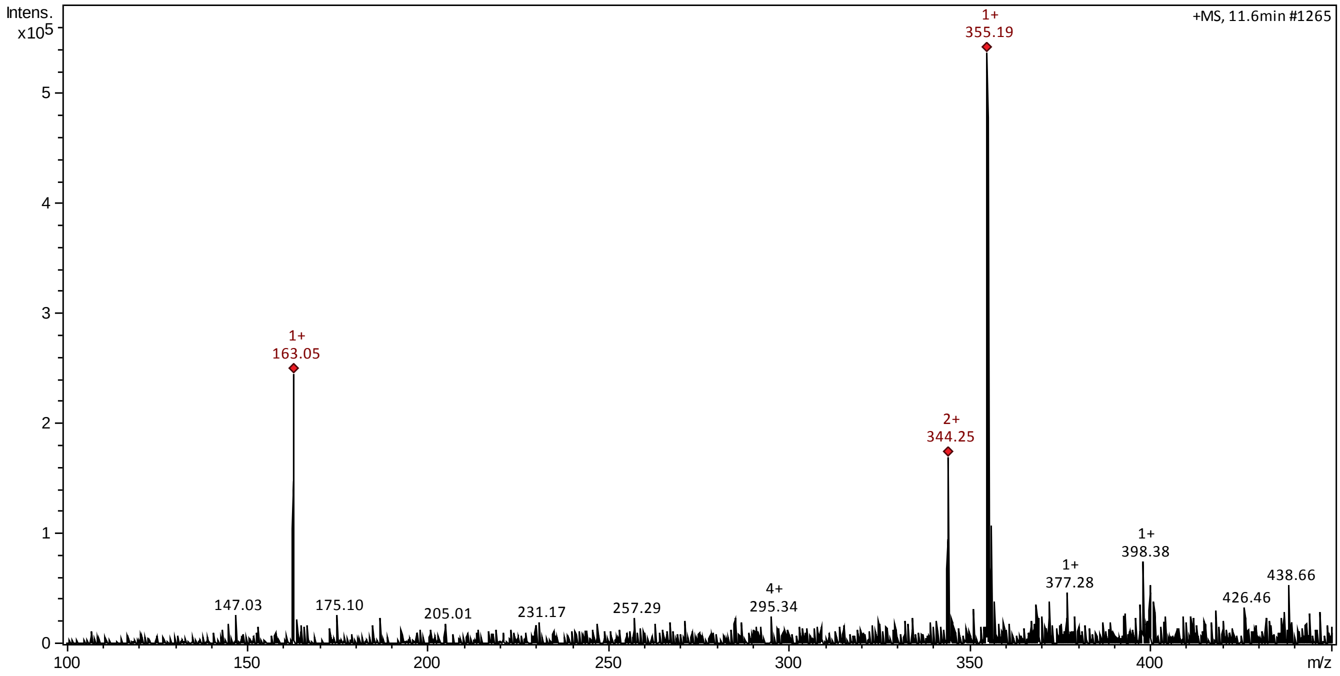

(+)-ESI-IT-MS/MS spectra of chrologenic acid (7) *m*/*z* 355.19 [M+H]$^+$

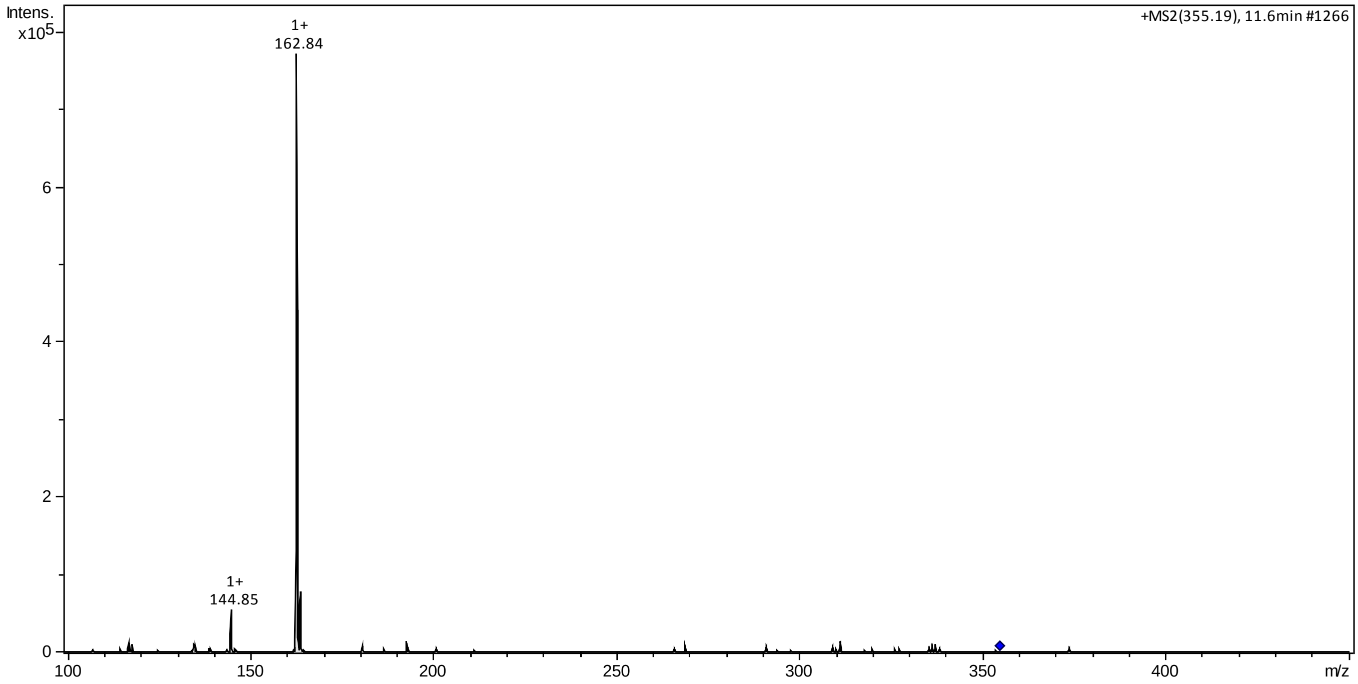

Supplement: Supplementary file 1 [file molecules-22-00910-s001.pdf]
